# DNA methylation, histone acetylation in the regulation of memory and its modulation during aging

**DOI:** 10.3389/fragi.2024.1480932

**Published:** 2025-01-06

**Authors:** Padmanabh Singh, Vijay Paramanik

**Affiliations:** Cellular and Molecular Neurobiology & Drug Targeting Laboratory, Department of Zoology, Indira Gandhi National Tribal University, Amarkantak, Madhya Pradesh, India

**Keywords:** DNA methylation, histone acetylation, learning and memory, aging, phytochemials

## Abstract

Memory formation is associated with constant modifications of neuronal networks and synaptic plasticity gene expression in response to different environmental stimuli and experiences. Dysregulation of synaptic plasticity gene expression affects memory during aging and neurodegenerative diseases. Covalent modifications such as methylation on DNA and acetylation on histones regulate the transcription of synaptic plasticity genes. Changes in these epigenetic marks correlated with alteration of synaptic plasticity gene expression and memory formation during aging. These epigenetic modifications, in turn, are regulated by physiology and metabolism. Steroid hormone estrogen and metabolites such as S-adenosyl methionine and acetyl CoA directly impact DNA and histones’ methylation and acetylation levels. Thus, the decline of estrogen levels or imbalance of these metabolites affects gene expression and underlying brain functions. In the present review, we discussed the importance of DNA methylation and histone acetylation on chromatin modifications, regulation of synaptic plasticity gene expression and memory consolidation, and modulation of these epigenetic marks by epigenetic modifiers such as phytochemicals and vitamins. Further, understanding the molecular mechanisms that modulate these epigenetic modifications will help develop recovery approaches.

## Introduction

The brain is the most complex and dynamic organ of an organism. It controls numerous vital functions, such as decision-making, cognition, learning, and memory. Memory is a higher-order brain function that stores and recalls previously acquired experiences, facts, and information. This acquired information is stabilized in the form of long-term memory by strengthening the synaptic connections, and it requires the expression of memory-linked synaptic plasticity genes (Kandel, 2001). The expression of these synaptic plasticity genes is altered during aging and neuropathological conditions, which affects learning and memory (Hermann et al., 2014). Previous research revealed that epigenetic mechanisms play an essential role in the regulation of these synaptic plasticity genes and dysregulation of which can lead to memory impairment during aging and age-associated neurodegenerative diseases such as Alzheimer’s disease (AD) and Parkinson’s disease (PD) (Singh et al., 2017).

## Epigenetic modifications

Chromatin is a complex interaction of DNA and histones that packs the large size of DNA inside the nucleus. The nucleosome is the smaller unit of the chromatin and is made of 147 bp DNA wrapped around a histone octamer core. The linker DNA is associated with a linker histone H1 (Handy et al., 2011). Chromatin is present in two states. During the condensed state, it inhibits the interaction of transcription factors on gene promoters and does not allow gene transcription. On the other hand, the relaxed state allows the interaction of transcription factors on gene promoters and allows gene transcription. The most important factors that regulate the condensation and relaxation of chromatin are the covalent modifications of DNA and histone proteins and thus influence gene expression (Lossi et al., 2024). The covalent modifications found on DNA are methylation and hydroxymethylation. Similarly, the most common post-translational modifications found on the n-terminal tail of histones are acetylation, phosphorylation, methylation, etc. These covalent modifications on DNA and histone alter their interactions in the nucleosome, thus regulating chromatin’s condensation and relaxation and thereby regulating gene expression (Kelly et al., 2010).

## DNA methylation

The methylation of DNA takes place on the cytosine (C) residue followed by a guanine (G) residue called CpG island and regulates gene expression at the transcriptional level. During this modification, a methyl (CH3) group is transferred from S-adenosyl methionine to the fifth carbon of cytosine (5 mC) in a CpG island (Gao et al., 2018). The transfer of methyl group on DNA is catalyzed by DNA methyltransferases (DNMTs). DNMTs are classified as *de novo* methyltransferase (DNMT3a and DNMT3b) which transfers a methyl group on a previously unmethylated CpG island on DNA and maintenance methyltransferase (DNMT1) which transfers a methyl group on a hemimethylated CpG site that arises as a result of DNA replication and maintains the DNA methylation pattern similar to pre-replication (Moore et al., 2013). The epigenetic mark, DNA methylation is found on the CpG sites on the promoter of a gene affecting the binding of transcription factors, and regulating gene expression. Also, the cytosine methylation on the CpG island at the promoter recruits a group of proteins called methyl-binding proteins. This interaction of cytosine methylation and methyl-binding proteins forms the repressor complex and suppresses gene transcription (Lu et al., 2013). DNA methylation also activates the expression of genes by recruiting activator complexes at their promoter region (Chahrour et al., 2008).

## DNA methylation and memory

Covalent modification of DNA regulates synaptic plasticity genes’ expression and underlying neuronal functions such as learning and memory. Levenson et al. (2004) first examined the role of DNA methylation and DNMTs in regulating synaptic plasticity and memory formation. To check the role of DNA methylation on synaptic plasticity gene expression, they treated hippocampal slices with DNMT inhibitor zebularine, and observed decreased CpG methylation levels at the promoter of reelin and BDNF. Further, the treatment of hippocampal slices with phorbol-12,13-diacetate, an activator of protein kinase C upregulated DNMT3a expression in the CA1 of the hippocampus. They also reported that zebularine or 5-aza-2-deoxycytidine, an inhibitor of DNA methylation, treatment impaired LTP in brain slice culture, which suggests that basal activity DNMTs are required for learning and memory.

To investigate further, reports from the same group checked the involvement of DNMTs during contextual fear memory. They observed that the expression of *de novo* DNMTs DNMT3a and 3b increased in the hippocampus 30 min post contextual fear paradigm in adult rats. However, the level of maintenance methyl transferase DNMT1 remained unchanged. The fear memory formation was impaired when DNA methylation was inhibited in these animals after contextual fear conditioning. Further, they checked the methylation level at the promoter of protein phosphatase 1 (PP1), which negatively regulates memory and reelin, which is important for learning and memory. Interestingly, they observed hypermethylation at the promoter of PP1 decreased its expression, and hypomethylation at the promoter of reelin increased its expression after fear memory formation. This suggests that DNA methylation marks are dynamic in nature and important for hippocampal-dependent memory formation during normal physiological condition (Miller and Sweatt, 2007).

Knockout studies in animal models showed that DNMTs play an important role in synaptic plasticity and memory formation. Conditional knockout (CKO) of DNMT1 in the forebrain of mice showed impaired barrel cortex development, the brain region important for processing touch inputs. Also, a patch clamp study showed impaired induction of LTP in DNMT1 KO brain slices, suggesting DNMT1 is important not only for development, however, also for synaptic plasticity (Golshani et al., 2005). CKO of DNMT1 and DNMT3a mice showed around 10% reduction in hippocampal volume and the volume of the neurons, mainly in the dentate gyrus region, with impaired LTP induction and enhanced LTD. DKO mice show impaired spatial memory and fear memory consolidation (Feng et al., 2010). Similarly, Liu et al. (2011) investigated the effect of DNMT1 haploinsufficiency in heterozygous DNMT1 ± mice during aging. They observed that the heterozygous DNMT1 ± mice showed lowered DNA methylation in the cerebral cortex and hippocampus during aging compared to age-match control. They observed a positive correlation between hypomethylation and impaired spatial memory during aging in DNMT1 ± mice as compared to the control.

## DNA methylation during aging and neurodegeneration

Several reports show that an imbalance of epigenetic changes, such as DNA methylation, is observed during aging and different neuropathological conditions (Table 1). Elsner et al. (2013) reported that the expression of DNMT1 but not DNMT3b declined in the hippocampus of old rats compared to young rats. Similarly, Singh and Thakur (2014) also reported that the level of DNMT1 but not DNMT3a and DNMT3b declined in the cortex and hippocampus of old compared to young and adult mice. Further, the decline of DNMT1 level is associated with impaired recognition memory in old mice. Mastroeni et al. (2011) reported that the expression of DNMT1 declined in Alzheimer’s disease (AD) patients as compared to age-matched healthy control. Further, the decline of DNMT1 level is associated with a reduction of total cytosine methylation in AD patients. In humans, DNMT1 mutation causes hereditary sensory and autonomic neuropathy (HSAN1). Mutation of DNMT1 leads to abnormal DNA methylation levels, resulting in neurodegeneration, hearing loss, and dementia in patients. At the molecular level, DNMT1 mutation causes aberrant heterochromatin formation during the G2 phase of the cell cycle, global-level hypomethylation, and local-level hypermethylation (Klein et al., 2011). Oliveira et al. (2012) observed that the Dnmt3a1 and Dnmt3a2 expression decreased in the hippocampus and cortex of old mice. CKO of DNMT3a2 reduced the expression of Arc, BDNF, Egr-1, and Nur-77 and hippocampal-dependent fear memory and recognition memory. Further, rescuing of DNMT3a2 expression improved cognitive functions in old mice. Further, age-dependent impairment in spatial memory consolidation is associated with increased promoter-specific methylation of ARC and EGR1 genes and decreased expression of synaptic plasticity genes in the CA1 and DG regions (Penner et al., 2011; Penner et al., 2016). Genome wide methylation studies showed that he global level of Cytosine methylation decreased in the hippocampus of AD patients as compared to age matched control (Chouliaras et al., 2013; Zocher, 2024). Similarly, decreased promoter level DNA methylation of APP and PSEN1 gene leads to their higher expression and Aβ formation in AD patients (West et al., 1995; Monti et al., 2020). PD is a neurodegenerative disease associated with the accumulation of α-synuclein (α-syn) protein (Negi et al., 2024). Analysis of *postmortem* brain sample of PD patients showed hypomethylation of SNCA gene promoter associated with upregulation of α-syn expression (Jowaed et al., 2010). On the other hand, promoter hypermethylation of PGC-1α associated with impaired mitochondrial biogenesis in the mice model of PD (Su et al., 2015).

**TABLE 1:** DNA methylation, DNMTs and their involvement in brain function and memory.

DNA methylation and DNMTs	Brain function and memory	References
DNMT1	Declined in the hippocampus during aging and associated with declined of hippocampal-dependent recognition memory. Upregulated in the hippocampus of amnesic mice and associated with declined of synaptic plasticity genes BDNF and Arc expression	Elsner et al. (2013) Singh and Thakur (2014) Srivas and Thakur (2017)
DNMT3a1 and 2	Declined in the hippocampus and cortex of old mice, regulate expression of synaptic plasticity genes Arc, BDNF, Egr-1, and Nur-77 and hippocampal-dependent fear and recognition memory	Oliveira et al. (2012)
Global DNA methylation	Decreased in the hippocampus of AD patients	Mastroeni et al. (2011) Chouliaras et al. (2013)
Increased Promoter Methylation	Increased promoter methylation of synaptic plasticity genes BDNF, NARP, Homer1, and EGR1, Reelin and associated with declined of hippocampal-dependent memory	Srivas and Thakur (2017) Singh et al. (2015) Ju et al. (2016)
Decreased Promoter Methylation	Decreased promoter level DNA methylation of APP and PSEN1 in AD patients and associated with higher Aβ formation	West et al. (1995) Monti et al. (2020)

Scopolamine is a M1 muscarinic antagonist used to induce amnesia animal models. Singh et al. (2015) reported that the expression of DNMT1 upregulated in the hippocampus of scopolamine-induced amnesic mice and associated with the decline expression of synaptic plasticity genes BDNF and Arc. Similarly, Srivas and Thakur (2017) also reported that the methylation level at the promoter of synaptic plasticity genes NARP, Homer1, and EGR1 increased, and their transcription decreased in the hippocampus of scopolamine-induced amnesic mice. Further, treatment of DNMTs inhibitor 5-aza-2′-deoxycytidine, reduced promoter level methylation of BDNF and Arc, upregulated their expression, and improved memory in amnesic mice. Sevoflurane is a general anesthetic that induces neurobehavioral abnormalities in rodents (Kameyama et al., 2022). Ju et al. (2016) reported that repeated exposure to sevoflurane decreased dendritic spines, impaired hippocampal-dependent spatial and fear memory in rats, and was associated with upregulation in the expression of DNMTs. Further, they observed that the downregulation of synaptic plasticity genes BDNF and Reelin was due to hypermethylation at their promoter regions. Further, pre-treatment of DNMTs inhibitor 5-AZA rescued the sevoflurane-induced memory impairment. These reports suggest that DNMTs are crucial during development and essential in regulating memory consolidation during aging and different physiological and pathological conditions.

## Histone acetylation

Histone proteins undergo several covalent modifications after the translation. These modifications include acetylation, phosphorylation, ubiquitination, methylation, etc. Among these, histone acetylation is correlated with active gene expression, while deacetylation leads to gene repression (Ramazi et al., 2020). Two groups of enzymes that regulate the acetylation level on the N terminal of the tail of histones are histone acetyltransferases (HATs) and histone deacetylases (HDACs). HATs transfer an acetyl group (−COCH₃) from the acetyl CoA to the histones and HDACs remove the acetyl group from the histones. Acetylation on histone reduces its interaction with DNA and results in relaxed or open chromatin, which allows the binding of transcription factors on the gene’s promoter region and, thus, active gene transcription (Clayton et al., 2006). On the contrary, deacetylation increases the interaction between histones and DNA, resulting in a closed or condensed chromatin, which inhibits the binding of transcription factors leading to gene repression. HATs are mostly transcriptional activators as they positively regulate gene expression. They are divided into the GNAT family, p300/CBP family, MYST family, Transcription-related HATs, and nuclear receptor-associated HATs (Selvi et al., 2010). The p300/CBP family is one of the vital histone lysine transferases and a transcription activator. This HAT family regulates many functions, including neurogenesis, neuronal development and differentiation, and memory formation (Partanen et al., 1999; Bedford and Brindle, 2012; Chatterjee et al., 2013; Alarcón et al., 2004; Barrett et al., 2011; Crump et al., 2011; Lopez-Atalaya et al., 2011). Deacetylases are classified into class I, II, III, and IV HDACs based on their localization in the cells. The class I includes HDAC1, 2, 3, and 8, and primarily present in the nucleus. They mostly regulate gene expression and act as transcriptional repressors. HDAC2 is one of the most important classes of HDACs studied extensively about the regulation of synaptic plasticity genes and memory (Kilgore et al., 2010).

## Histone acetylation and memory

CREB binding protein (CBP) is an important HAT that performs its role as a transcriptional activator of many synaptic plasticity genes and helps in memory consolidation. Mice with CBP CKO showed normal neuronal morphology but lowered histone acetylation levels and reduced long-term associative and recognition memory formation. Further, they reported downregulating synaptic plasticity genes such as NMDARs, AMPARs, PSD95, etc (Chen et al., 2010). CKO of CBP in the forebrain of mice reduced histone acetylation at H2A, H2B, H3, and H4 and impaired recognition memory (Valor et al., 2011). Similarly, mice with CKO of CBP in the medial prefrontal cortex showed impaired long-term spatial, object location, and fear memory (Vieira and Korzus, 2015; Korzus et al., 2004). Guan et al. (2009) reported that HDAC2 overexpression decreased spine density, histone H3K14/K12 acetylation, negatively regulated synaptic plasticity gene expression such as BDNF, GluR, EGR1, etc., and impaired memory consolidation. However, no changes in synaptic plasticity gene expression, and memory were observed when HDAC 1 was overexpressed. Hsiao et al. (2017) reported that HDAC2 knockout APP/PS1 mice showed improved freezing behavior. Further, the binding of HDAC2 decreased at the promoter of synaptic plasticity gene BDNF which results increased BDNF gene expression. This showed that HDAC2 is an important regulator of gene expression and learning, and memory.

Histone acetylation is dependent on neuronal activity and is essential for regulating synaptic plasticity gene expression and memory consolidation (Table 2). Levenson et al. (2008) reported that acetylation on H3 is essential for long-term hippocampal-dependent memory formation. Fischer et al. (2007) reported that acetylation at H3K9/14 and H4K5/8/12 in the hippocampus increased upon environmental enrichment and is associated with improved memory consolidation in CK-p25 mice. Acetylation on the cortex and hippocampus is very dynamic. Gräff et al. (2012) reported that acetylation at H3K14 and H4K5 increased rapidly and transiently in the hippocampus however increased slowly and persistently in the cortex after a novel object recognition test. This difference in acetylation pattern may be due to their difference in functions during memory consolidation.

**TABLE 2:** List of histone acetylation and their involvement in learning and memory.

Histone acetylation	Function in memory	References
H3K9 Ac	Recovered spatial and associative memory in a neurodegenerative mouse, recovered recognition memory in an amnesic mouse, enhanced fear memory	Fischer et al. (2007) Lubin and Sweatt (2007) Singh et al. (2015)
H3K14 Ac	Recovered spatial and associative memory in a neurodegenerative mouse, recovered recognition memory in an amnesic mouse, enhanced object recognition memory, enhanced contextual fear conditioning memory	Fischer et al. (2007) Lubin and Sweatt (2007) Gräff et al. (2012) Singh et al. (2015)
H4K5 Ac	Recovered spatial and associative memory in neurodegenerative mouse	Fischer et al. (2007)
H4K8 Ac	Recovered spatial and associative memory in a neurodegenerative mouse, enhanced object location memory due to exercise, enhanced object recognition and location memory, enhanced spatial memory	Fischer et al. (2007) Gräff et al. (2012) Intlekofer et al. (2013)
H4K12 Ac	Recovered spatial and fear memory associative memory in neurodegenerative mice, recovered of fear memory in old	Fischer et al. (2007) Gräff et al. (2012)

## Histone acetylation during aging and neurodegeneration

Total histone acetylation level and gene promoter-specific histone level decreased during aging and neurodegenerative diseases. This change in acetylation level is a result of alteration in HATs and HDACs expression. Chung et al. (2002) reported that the CBP immunoreactivity decreased significantly in the cerebral cortex and the CA1 and DG region of the hippocampus in old rats as compared to the adult. Further, the decline of CBP is mainly observed in the pyramidal layer as well as in the granule cell layer and the polymorphic layer of CA1/CA3 and DG region of the hippocampus respectively. Giralt et al. (2012) reported that the expression of CBP decreased and was associated with lowered acetylation level at H3 and memory impairment in HD mice model. Further, treatment of C646, a selective inhibitor of p300/CBP, decreased histone acetylation level and impaired recognition and fear memory consolidation (Mitchnick et al., 2016; Maddox et al., 2013). On the other hand, CBP over-expression rescued spatial memory and increased the expression of BDNF in the AD mouse model (Caccamo et al., 2010). CBP positively regulates several synaptic plasticity gene expressions such as ARC, BDNF, c-FOS, EGR-1, etc., and thereby helps in memory consolidation. On the other hand, the downregulation of CBP is associated with the downregulation of these synaptic plasticity genes and memory impairment (Wood et al., 2005; Wood et al., 2006).

Gräff et al. (2012) reported that the expression of HDAC2 increased in the brains of AD patients and the hippocampus of CK-p25 mice, a mouse model of neurodegeneration. Further, the knockdown of HDAC2 rescued histone acetylation, ARC, BDNF, and EGR1 expression and memory consolidation in CK-p25 mice. Similarly, Singh and Thakur (2014) reported that HDAC2 level upregulated in the hippocampus and negatively correlated with recognition memory consolidation in old mice. Reports from the same group also showed that upregulation of HDAC2 is also associated with reduced acetylation levels at the promoter of BDNF and Arc. Inhibition with sodium butyrate (NaB) or knockdown of HDAC2 in the hippocampus, rescued histone acetylation level, gene expression, and memory (Singh and Thakur, 2018). Singh et al. (2015) reported that the level of HDAC2 was upregulated in the hippocampus of scopolamine-induced amnesic mice and negatively regulated synaptic plasticity gene expression and hippocampal-dependent memory consolidation. Aging, neurodegeneration, and amnesic conditions are associated with decreased total histone acetylation levels at H3 and H4 as well as at the promoter of synaptic plasticity genes and thus memory impairment (Gräff et al., 2012; Singh et al., 2015; Singh and Thakur, 2018).

Several reports showed that acetylation at H3K9 and H3K27 increased in the brain of AD patients and upregulated the expression of AD related gene and underlying neurodegeneration (Marzi et al., 2018; Klein et al., 2019; Nativio et al., 2020). Similarly, Nativio et al. (2018) reported that acetylation at H3K16 position decreased in the brain of AD patients as compared to cognitively normal aged individuals. Dysregulation of histone acetylation is also observed in the PD patients. Genome wide acetylation analysis in PD *postmortem* brain sample showed increased H3K27 acetylation and higher expression of gene associated with PD pathology such as SNCA, MAPT and PRKN (Toker et al., 2021). Similarly, higher histone acetylation level was observed in the brain sample of PD patients as well as cellular model of PD and MPTP treated mice brain. This increased in histone acetylation in PD brain samples associated with decreased HDAC1,2 and 6 (Park et al., 2016). In a comparison study between APP/PS1 and wild type mice during aging, McClarty et al. (2024) reported that impaired recognition and spatial memory in old (18 months) wild type mice and mid aged (12 months) and old (18 months) APP/PS1 mice associated with increased HDAC2 in the hippocampus and HDAC3 in the pre frontal cortex. This increased in HDACs decreased H3K9 acetylation level and synaptic plasticity gene expression. On the other hand, different behavioral paradigms such as environmental enrichment, contextual fear conditioning, and object location memory is associated with increased total histone acetylation at H3 and H4 as well as at the promoter of synaptic plasticity genes during different physiological conditions such as aging, neurodegeneration, etc. (Intlekofer et al., 2013; Lubin and Sweatt, 2007; Fischer et al., 2007; Wang et al., 2020).

## Regulation of DNA methylation and histone acetylation

These epigenetic marks on DNA and histones are modulated by physiology and metabolism. Folate/vitamin B12 pathway, acetyl CoA metabolism as well as hormone-like estrogen 17β-estradiol (E2) plays important roles in regulating DNA methylation and histone acetylation (Fortress et al., 2014; Frick et al., 2015). Alteration in metabolic pathways and hormonal levels during aging and neurodegenerative diseases affects DNA methylation and histone acetylation levels and, thus, gene expression and underlying brain functions (Singh and Paramanik, 2022).

The methyl group transferred to the CpG is donated by S-adenyl-methionine (SAM). The synthesis of SAM is catalyzed by methionine adenosyltransferase enzymes using the amino acid methionine and ATP (Cantoni, 1953). After removal of a methyl group, SAM is converted to S-adenosyl-homocysteine (SAH) and finally to homocysteine. This homocysteine is then recycled methionine by a vitamin B12-dependent enzyme methionine synthase with the help of 5-methyltetrahydrofolate, a folate derivative. Thus, methionine, vitamin B12, and folate are essential to maintain the methylation level. SAH is a potent inhibitor of DNMTs, therefore, accumulation of SAH affects the DNA methylation process. In a long-term population-based study, Mihara et al. (2022) reported that a high SAM/SAH ratio is associated with a lower risk of dementia and death. Using a folate and vitamin B12-deprived media to mimic AD-like conditions, Fuso et al. (2005) reported that the methylation at the promoter of APP and Presenilin1 decreased, and their expression increased in neuroblastoma cell lines. The upregulation of APP and Presenilin1 leads to the production of higher amyloid β levels. Further, administration of SAM in the deprived media reversed promoter methylation, APP, and Presenilin1 expression, and the reduced amyloid β level. Similarly, they also showed that deprivation of vitamin B12, B6, and folate dietary deficiency leads to hyperhomocysteinemia due to alteration of SAM and SAH level in TgCRND8 and 129Sv mice. The imbalance of SAM and SAH is associated with the upregulation of PS1 and BACE with higher amyloid β deposition and cognitive impairment (Fuso et al., 2008). This showed that an imbalance in SAM and SAH levels decreased methylation at the promoter of APP, PS1, and BACE that leads to higher amyloid β deposition, a characteristic feature of AD. To check the effect of early-life SAM supplementation on AD symptoms in an AD mice mouse model, Raia et al. (2023) reported that perinatal supplementation of SAM repressed PS1 expression and amyloid β deposition in adult AD mice. Further, the effect of perinatal SAM supplementation is similar to long-term post-weaning supplementation of SAM regarding AD symptom manifestation in adult AD mice. This showed early life SAM supplementation is beneficial and can lead to healthy aging.

The acetyl group on histone acetylation is donated by Acetyl CoA, a product of glucose metabolism. Metabolic enzymes like Pyruvate dehydrogenase complex (PDC) and ATP citrate lyase (ACL) regulate the synthesis of Acetyl CoA and are thus involved in chromatin remodelling and gene expression (Brinton, 2008; Biju et al., 2024). Wellen et al. (2009) reported that ATP citrate lyase (ACL), a key metabolic enzyme that converts citrate into acetyl-CoA in the cytoplasm and is an essential link between cellular metabolism and histone acetylation level in the nucleus. Further, the metabolic enzyme PDC consists of three enzymes Pyruvate Dehydrogenase (E1), Dihydrolipoyl transacetylase (E2), and Dihydrolipoyl dehydrogenase (E3) is first to convert pyruvate into Acetyl CoA in the mitochondria which then used in the citric acid cycle to generate ATP and Citrate which ACL then uses to synthesize Acetyl CoA, Acetyl group donor for histone (Choudhary et al., 2014). Alterations in the expression and activities of these metabolic enzymes are found in aging and neurodegenerative diseases. Perry et al. (1980) reported that the ACL and PDC activity decreased in the post-mortem brain tissue of AD patients. Similarly, Sorbi et al. (1983) observed that PDC activity decreased the brain of AD and Huntington disease patients. Further, Wellen et al. (2009) established that ATP-citrate lyase and citrate are essential for metabolism and histone acetylation. Jiang et al. (2008) elucidated the metabolic profile in SAMP8 mice, a rodent model, to study age-associated memory impairment and neurodegeneration. They reported that the level of many metabolites that are responsible for cellular metabolism, including citrate and pyruvate, is reduced in the serum. Further, the alteration of these metabolites is higher in females than males. This may be one reason that females are more vulnerable to neurodegenerative diseases. Peleg et al. (2010) also reported that the citrate level is crucial for nuclear histone acetylation and gene expression. The reduction of citrate level impaired histone acetylation level, gene expression, and memory in old age. Short-chain fatty acid such as butyrate is synthesized as a result of the fermentation of dietary fiber by the microbes in the intestine. Further, dietary supplementation of high fiber diet or butyrate producing microbe improve cognitive functions, reduced anxiety and stress as compared to low fiber diet in human subjects and animal models (Khan et al., 2015; Bourassa et al., 2016). In a “MitoPark” PD mice model, mitochondrial stress associated with increased H4K12 acetylation and degeneration of DAergic neurons. This change in H4K12 acetylation level is due to imbalance of HATs and HDACs (Huang et al., 2024).

Sex steroid hormone, such as estrogen 17β-estradiol (E2), plays a crucial role in learning and memory by regulating hippocampal neuronal morphology, plasticity, and memory in different models, and its decline after menopause severely increases cognitive declined and chance of neurodegenerative during aging (Frick, 2009; Pike et al., 2009; Viña and Lloret, 2010; Zárate et al., 2017; Singh and Paramanik, 2022). Initial research in the field of E2-mediated epigenetic regulation showed E2-induced DNA demethylation and histone acetylation in the brains of young and adult rats as compared to old rats (Thakur et al., 1978; Thakur and Kanungo, 1981). These changes in DNA methylation and histone acetylation were associated with higher gene transcription in young and adults when compared to old rats (Kanungo and Thakur, 1979). Estrogen-mediated signaling pathways help mitochondria to enhance aerobic respiration through the coupling of glycolysis to the Krebs cycle and ATP synthesis in hippocampal and cortical neurons. Apart from mitochondrial function and bioenergetics, E2 regulates the expression of enzymes like DNMTs, HDAC2, CBP, PDC, and ACL and thus indirectly regulates chromatin remodelling and gene expression (Pearce and Balnave, 1976; Brinton, 2008). Zhao et al. (2010) observed that hippocampal administration of E2 increased the expression of DNMT3a and DNMT3b and improved recognition memory in mice. Further, this effect of E2 was diminished when mice co-treated with 5-aza-2′-deoxycytidine. Further, they also reported that intrahippocampal administration of E2 decreased HDAC2 expression, increased H3/H4 acetylation level, and improved recognition memory. Thus, E2 plays an important role in regulating histone acetylation modifications and synaptic plasticity gene expression and improves cognitive performance in aged as well as AD mouse models (Frick et al., 2002; Heikkinen et al., 2004; Frye et al., 2005).

## Therapeutic potential of epigenetic modifiers

Epigenetic modifications such as DNA methylaton and histone acetylation are reversible in nature. Therefore, these modifications are suitable for drug targeting. Several drugs, small molecules as well as plant derived molecules and herbal formulations are known to be target chromatin modifying enzymes and underlying epigenetic modifications.

Phytoestrogens are a group of plant derived compounds having similar chemical structure to steroid hormone estrogen. Similar to estrogen, phytoestrogens also shown to modulate the DNA methylation and histone PTMs. Genistein is a plant polyphenols, most abundantly found in soy and soy-based product. Due to its structural similarities with estrogen, genistein is also known as phytoestrogen. Studies in animal model suggests that genistein is neuroprotective in nature and help in learning and memory. Bagheri et al. (2012) reported that genistein treatment reduced the Aβ_1-40_ plaque formation in the hippocampus and improved learning and memory in AD rat model. Khodamoradi et al. (2017) reported that intraperitoneal administration of soy extract containing genistein improved long term potentiation, learning and memory in ovariectomized rats as compared to control rats. Studies in different cancer cell line suggests geneistein also found to be regulate epigenetic modification such as DNA methylation and histone acetylation. Genestein treatment alters the expression of chromatin modifying enzymes. The expression of DNMTs (DNMT1, DNMT3a and DNMT3b) and HDACs (HDAC1, HDAC5 and HDAC6) downregulated while HATs (CIITA and ESCO2) upregulated in geneistein treated HeLa cells. Also geneistein treatment decreased the activity of DNMTs and HDACs as well as global DNA CpG methylation level (Sundaram et al., 2019). Geneistein treatment decreased DNMT activity, DNMT1 expression and global DNA methylation. In slilico analysis showed genestein inhibit the activity of DNMT1 by interacting directly with its catalytic domain Xie et al. (2014).

Epigallocatechin-3-gallate is a polyphenols found in tea. Fang et al. (2007) reported that Epigallocatechin-3-gallate inhibits DNMTs that leading to promoter specific hypomethylation and increased gene expression in esophageal cancer cell line. Apart from acts as DNMT inhibitor, Epigallocatechin-3-gallate also inhibits HDACs and decreased the expression of APP in neuronal cell (Hu et al., 2015). Epigallocatechin-3-gallate treatment inhibited HDAC1 activity, downregulated APP expression and decreased Aβ level in AD mice model (Chang et al., 2015). Curcumin is polyphenolic compound found in the turmaric. Due to its anti-inflammatory and antioxidant properties turmaric is commonly used in tradiational Indian ayurvedic medicine (Hatami et al., 2019; Sharifi-Rad et al., 2020). Apart from these, curcumin also modulates chromatin modifying enzymes and underlying gene expression (Abdul-Rahman et al., 2024). Several studies showed that curcumin inhibits DNMTs activity and expression; and thus decreased methylation level in cancer cell (Liu et al., 2009; Yu et al., 2013; Hassan et al., 2015). Lu et al. (2014) reported that curcumin inhibits HAT/P300, decreased promoter level histone H3 acetylation and the expression of PS1 and BACE in cellular model of AD. Dysregulation of SAM level altered expression and accumulation of APP and phosphorylated Tau, which in turn increased Aβ secretion in AD cellular model (Sontag et al., 2007). Similarly, Fuso et al. (2007) reported that folic acid, vitamin B12 and B6 deprivation reduced SAM level and increased AD related genes and Aβ secretion in neuroblastoma cell line. Further, supplementation SAM reverse the AD related gene expression and Aβ secretion. Lee et al. (2024) reported that supplementation of folic acid and vitamin B12 for 6 months improved Mini-Mental State Examination scored in AD patients as compared to controls. These result suggest phytochemicals and small molecules regulate expression and activities of chromatin modifying enzymes and plays important role in recovery of brain functions and learing and memory in during aging and associated pathologies.

## Conclusion

**FIGURE 1:**
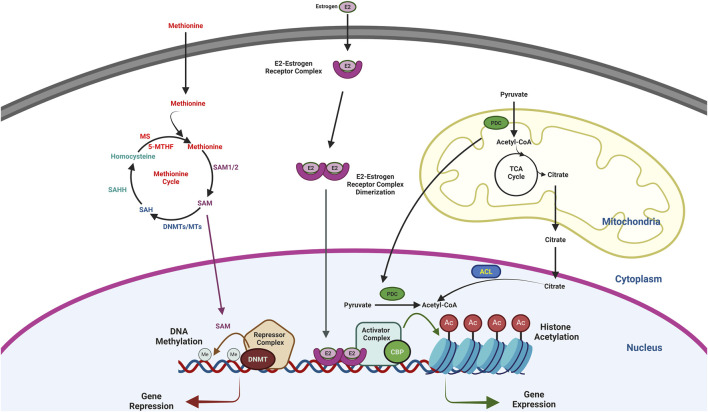
Schematic diagram showing the regulation of gene expression by DNA methylation and histone acetylation. DNMT, a part of the repressor complex methylates DNA that inhibit gene expression. On the other hand, CBP, a part of activator complex acetylates histone that activate gene expression. SAM, which is the substrate of DNA methylation is a product of Methionine cycle. After donating the methyl group, SAM converted to SAH and then homocysteine to continue the metheonine cycle. Further, Acetyl-CoA, the substrate for the histone acetylation is mainly synthesized by PDC in the nucleus and ACL by using the citrate, a product of TCA cycle in the mitochondria. Therefore, SAM and Acetyl-CoA play an important role in regulating the epigenetic modifications and gene expression. E2 also plays a crucial role in regulating histone acetylation and gene expression. E2-ER dimer after translocate to the nucleus recruits the activator complex and helps in histone acetylation and gene expression.

Due to their dynamic nature, DNA methylation and histone acetylation levels alter during different physiological conditions including aging, which inhibits gene expression and impaired synaptic plasticity. Reports also showed that these epigenetic marks modulated by physiology and metabolic processes and dietary supplementation epigenetic modifiers are beneficial in improving chromatin modifications and brain functions (Figure 1). Growing evidence showed the presence of PDC in the nucleus, synthesizes Acetyl CoA, and donates the acetyl group for histone acetylation. This nuclear PDC is translocated directly from the mitochondrial matrix to the nucleus (de Boer and Houten, 2014; Ng and Tang, 2014; Sutendra et al., 2014). Therefore, it will be essential to study the translocation of PDC from mitochondria as mitochondria are mostly affected during aging and neurodegenerative diseases. Further, these epigenetic marks, gene expression, and memory can be reversed by applying enzyme inhibitors. Phytochemicals such as phytoestrogens, polyphenols and as well as B vitamins plays important role in regulating the expression and activities chromatin modifying enzymes, global and promoter level DNA methylation, histone acetylation and gene expression. Further, many phytochemicals and small moleules also showed chromatin modifying activities but they have not been explored in brain or neuronal cells. As chromatin-modifying enzymes regulate diverse functions apart from regulating synaptic plasticity, the application of these inhibitors showed many side effects (Subramanian et al., 2010; Zhang et al., 2022). Thus, the designing of specific inhibitors is also needed apart from supplementation to rescue epigenetic dysregulation and memory during neurodegenerative diseases.
